# Importance of Per2 in cardiac mitochondrial protection during stress

**DOI:** 10.1038/s41598-024-51799-w

**Published:** 2024-01-14

**Authors:** Meghana Bhaskara, Olufisayo Anjorin, Arris Yoniles, Jianyun Liu, Meijing Wang

**Affiliations:** 1 Indiana University School of Medicine, Indianapolis, IN USA; 2 Department of Surgery, Indiana University School of Medicine, 950 W. Walnut Street, R2 E319, Indianapolis, IN 46202 USA

**Keywords:** Cardiology, Medical research

## Abstract

During myocardial injury, inflammatory mediators and oxidative stress significantly increase to impair cardiac mitochondria. Emerging evidence has highlighted interplays between circadian protein—period 2 (Per2) and mitochondrial metabolism. However, besides circadian rhythm regulation, the direct role of Per2 in mitochondrial performance particularly following acute stress, remains unknown. In this study, we aim to determine the importance of Per2 protein’s regulatory role in mitochondrial function following exposure to inflammatory cytokine TNFα and oxidative stressor H_2_O_2_ in human cardiomyocytes. Global warm ischemia (37 °C) significantly impaired complex I activity with concurrently reduced mitochondrial Per2 in adult mouse hearts. TNFα or H_2_O_2_ decreased Per2 protein levels and damaged mitochondrial respiratory function in adult mouse cardiomyocytes. Next, mitochondrial membrane potential ($\Delta \psi$_M_) using JC-1 fluorescence probe and mitochondrial respiration capacity via Seahorse Cell Mito Stress Test were then detected in Per2 or control siRNA transfected AC16 Human Cardiomyocytes (HCM) that were subjected to 2 h-treatment of TNFα (100 ng/ml) or H_2_O_2_ (100 μM). After 4 h-treatment, cell death was also measured using Annexin V and propidium iodide apoptosis kit through flow cytometry. We found that knockdown of Per2 enhanced TNFα-induced cell death and TNFα- or H_2_O_2_-disrupted $\Delta \psi$_M_, as well as TNFα- or H_2_O_2_-impaired mitochondrial respiration function. In conclusion, Per2 knockdown increases likelihood of cell death and mitochondrial dysfunction in human cardiomyocytes exposed to either TNFα or H_2_O_2_, supporting the protective role of Per2 in HCM during stress with a focus on mitochondrial function.

## Introduction

The heart is more susceptible to ischemic injury compared to other solid organs because of its innately high metabolic demands. Mitochondria, the organelle vital to cardiomyocytes^1^, continuously supply oxidative energy to maintain heart function. During myocardial injury, mitochondrial impairment is an essential determinant of cardiomyocyte damage due to impaired mitochondrial ATP synthesis; increased generation of reactive oxygen species (ROS)^2^; inability to maintain mitochondrial membrane potential; decreased ion balance maintenance, particularly Ca^2+^; and increased mitochondrial permeability.

Accumulating evidence has highlighted interplays between the circadian rhythm and mitochondrial metabolic pathways^3–9^. Particularly, period circadian regulator 2 (Per2) is reported to control mitochondrial oxidative metabolism in mouse skeletal myoblasts^3^ and lipid metabolism in adipogenesis^6^. Per2 knockout (KO) worsens cold treatment (4 °C)-lowered triglyceride levels in brown adipocytes and reduces β-oxidation in brown adipose tissue mitochondria^7^. Per2 is also involved in metabolic regulation in the heart during ischemic damage^5,8^. These findings suggest a role for Per2 in the regulation of mitochondrial metabolic activity. However, Per2’s regulatory activity in mitochondrial metabolism in these studies is contingent upon the central nervous system’s response to stress. Cyclical production and activity of Per2 in the suprachiasmatic nucleus, midbrain, and forebrain is responsible for the activation of central clock pathways and subsequent metabolic adaptations of whole body and peripheral organs and tissues. Still, the direct role of Per2 in mitochondrial performance, besides circadian rhythm, remains unknown.

Our most recent study identified Per2 as a significantly down-regulated gene with upstream regulator activity that was inhibited in adult mouse hearts after 6 h-cold storage ex vivo^10^. Its downregulation corresponded with mitochondrial dysfunction. Knockdown of Per2 was also observed to impair mitochondrial membrane potential in H9c2 cells following cold storage^10^. Furthermore, hypoxia preconditioning increased Per2 translocation into the mitochondria and induced binding of Per2 to Complex IV in endothelial cells^11^. These findings imply a suspected role of Per2 in directly impacting mitochondrial function beyond circadian rhythm activities.

During a variety of acute myocardial injury, TNFα and H_2_O_2_ (one of the most stable ROS forms) rise markedly proceeding cardiac damage and dysfunction^12–19^. Local increase in inflammatory mediators and oxidative stress have been shown to impair cardiac mitochondria. In this study, we aim to determine the importance of Per2 protein’s regulatory role in mitochondrial function following exposure to inflammatory cytokine TNFα and oxidative stressor H_2_O_2_ in human cardiomyocytes.

## Results

### Stress-induced mitochondrial dysfunction is associated with reduced Per2 levels in the heart mitochondria and primary cardiomyocytes

**Figure 1:**
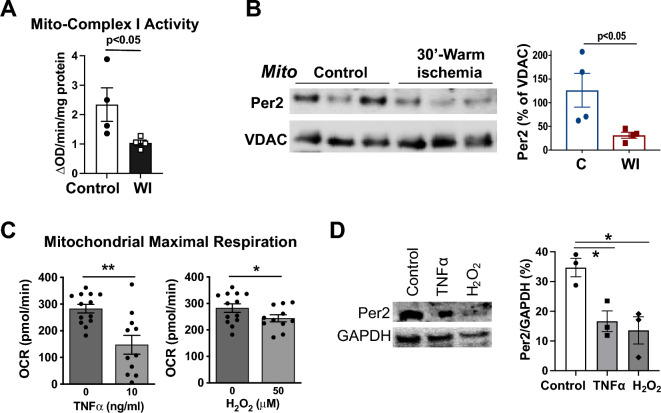
Stress-induced mitochondrial dysfunction is associated with reduced Per2 levels in mouse heart mitochondria and cardiomyocytes. (**A**) Ischemia causes cardiac mitochondrial dysfunction. Mitochondria (mito) isolated from adult mouse hearts ± 30 min-warm ischemia (WI) were used for detection of mito-complex I activity determined by a microplate reader. (**B**) Mitochondrial (Mito) Per2 content in mouse hearts ± 30-min WI. (**C**) TNFα or H_2_O_2_ treatment reduces mitochondrial maximal oxygen consumption rate (OCR). Mitochondrial Maximal Respiration = Maximal OCR after using FCCP—nonmitochondrial OCR after addition of rotenone (R) and antimycin A (A) in Fig. S2. (**D**) Per2 protein levels in mouse adult cardiomyocytes ± 2 h-TNFα (10 ng/ml) or -H_2_O_2_ (50 μM) treatment. Mean ± SEM, *p < 0.05, **p < 0.01.

Mitochondrial damage is evident following myocardial ischemia. In this study, we first confirmed that the activity of mitochondrial complex-I was severely impaired following ischemia in mitochondria isolated from adult male mouse hearts ± 30-min ischemia (Fig. 1A). Interestingly, we observed that ischemia decreased mitochondrial Per2 content in the heart compared to controls (Fig. 1B). Our previous study has demonstrated purity of the mitochondrial preparation, confirming no cytosolic and nuclear protein contamination^20^. Per2 may impact mitochondrial function independent of its subcellular location. Therefore, we mainly utilized the whole cell Per2 instead of mitochondria in this study. To emulate the high levels of TNFα or H_2_O_2_ induced early by myocardial ischemia, we then utilized TNFα or H_2_O_2_ as stressors in mouse adult cardiomyocytes and assessed the resulting mitochondrial metabolic function. We found that TNFα and H_2_O_2_ individually impaired maximal respiratory capacity in cardiomyocyte mitochondria (Fig. 1C), which was associated with decreased Per2 levels (Fig. 1D). Given that mitochondrial fraction of Per2 is involved in the circadian rhythm-controlled glycolysis-related energy pathway^21^, our results suggested a possible role of Per2 in protecting cardiac mitochondrial function during stress.

### Per2 expression impacts mitochondrial membrane potential (∆ψ_M_) in AC16 human cardiomyocytes subjected to TNFα or H_2_O_2_

**Figure 2:**
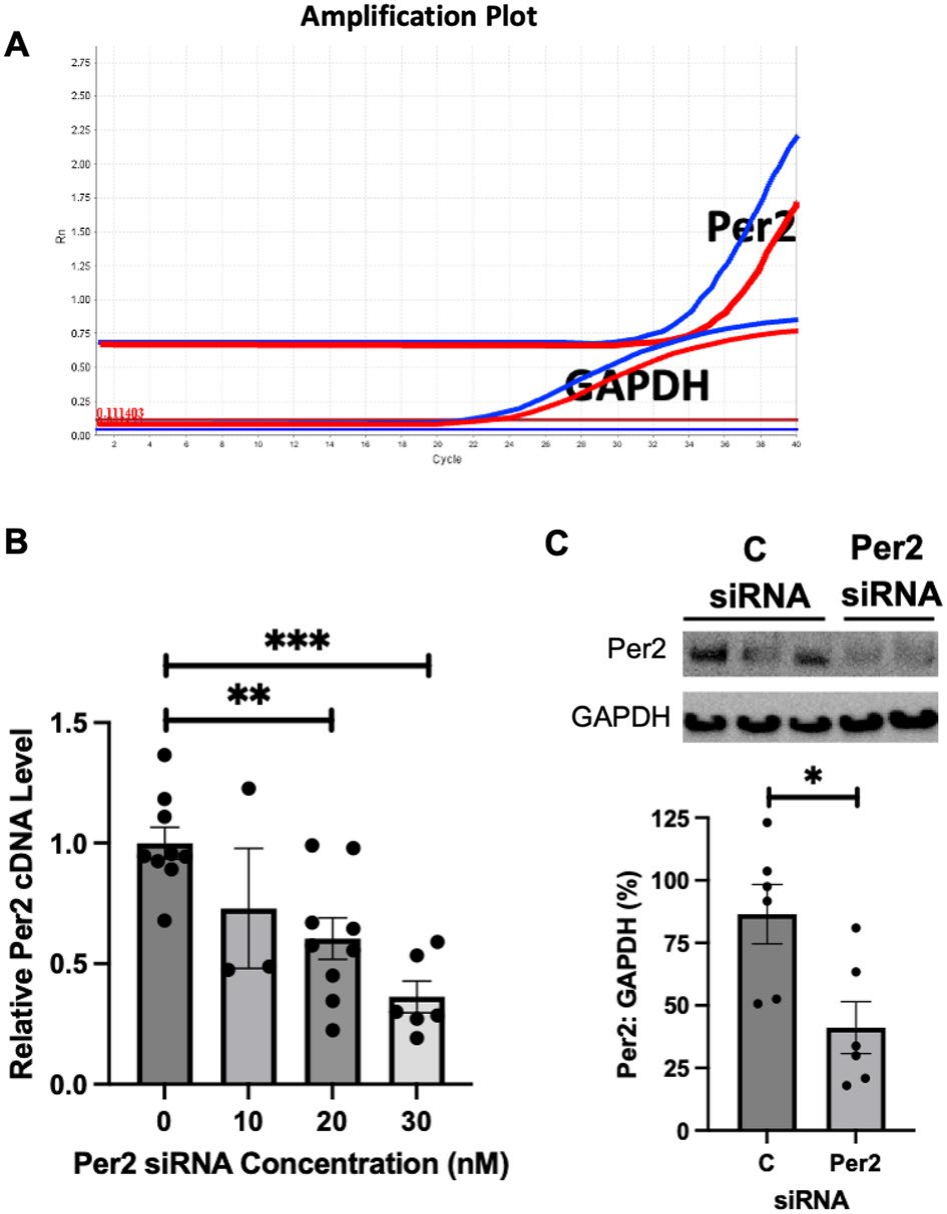
Per2 level is reduced by Per2 siRNA in AC16 human cardiomyocytes. (**A**) Representative amplification plots of Per2 and GAPDH with control (blue) vs. Per2 siRNA (red) transfection. (**B**) Dose-dependent Per2 mRNA knockdown with siRNA transfection. (**C**) Protein levels of Per2 in AC16 cells transfected with Per2 siRNA compared to control siRNA group. Mean ± SEM, *p < 0.05, **p < 0.01, ***p < 0.001.

To evaluate the role of Per2 in regulating mitochondrial vulnerability during stress, we then employed siRNA to knockdown Per2 expression in AC16 cells (human cardiomyocyte cell line). Our data showed a dose-dependent decrease in Per2 mRNA transcripts, with 10 pmol of Per2 siRNA resulting in the smallest reduction of Per2 mRNA compared to control levels, whereas 30 pmol of Per2 siRNA treatment resulted in the greatest reduction (Fig. 2B). We selected a dose of 20 pmol Per2 siRNA in this study, as higher doses of siRNA transfection are injurious to cells, but this dose still induced a significant reduction in Per2 mRNA (Fig. 2B). Western blot analysis further confirmed Per2 protein knockdown with 20 pmol of Per2 siRNA usage in AC16 cells (Fig. 2C).

**Figure 3:**
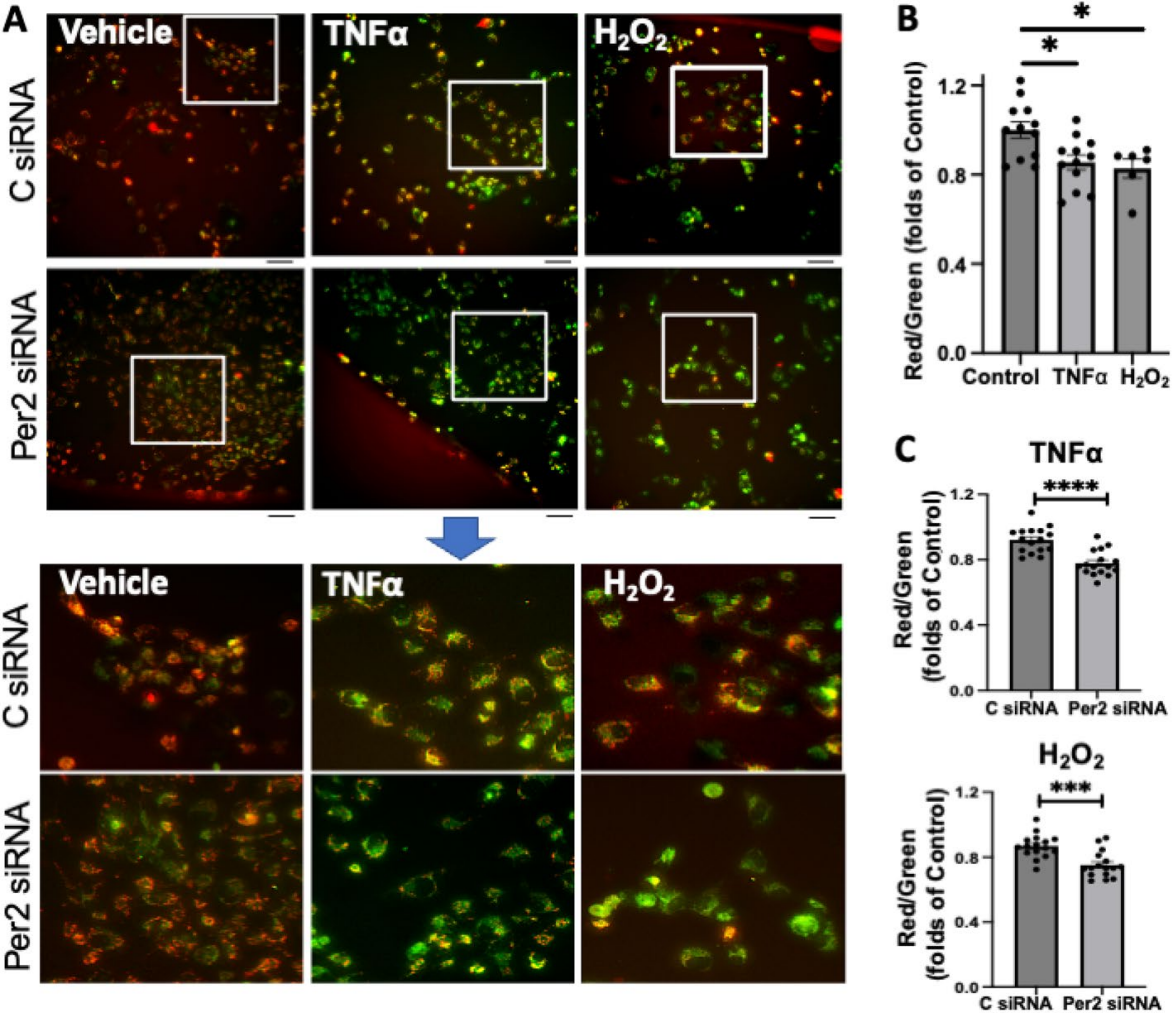
Per2 expression impacting mitochondrial membrane potential ($\Delta \psi$_M_) in AC16 human cardiomyocytes subjected to TNFα or H_2_O_2_. (**A**) Representative images of mitochondrial $\Delta \psi$_M_ using JC-1 in control (C) or Per2 siRNA-transfected AC16 cells after 2 h treatment of TNFα or H_2_O_2_.Scare bar = 100 μM. (**B**) TNFα and H_2_O_2_ decreased $\Delta \psi$_M_ in AC16 cells, represented as folds of the control group. (**C**) Per2 Knockdown potentiated $\Delta \psi$_M_ reduction in AC16 cells treated with TNFα and H_2_O_2_, represented as folds of the control in C siRNA or Per2 siRNA group, respectively. Red and green fluorescence intensity in each well was obtained using a microplate reader. The red to green fluorescence intensity ratio was analyzed to indicate $\Delta \psi$_M_ in (**B**) and (**C**). Mean ± SEM, *p < 0.05, ***p < 0.001, ****p < 0.0001. Dot represents individual well.

Mitochondrial membrane potential (*∆ψ*_***M***_) is critical for maintaining mitochondrial function and health. We first assessed mitochondrial membrane potential using JC-1 fluorescent probe in TNFα- or H_2_O_2_-stressed AC16 cells with control or Per2 siRNA transfection. JC-1 displays green fluorescence in the monomeric form in the cytosol upon depolarization, whereas it shows red fluorescence in the aggregated form in active mitochondria. Thus, the ratio of red/green fluorescent intensity indicates mitochondrial membrane potential. In addition to live-cell imaging on AC16 cells using a fluorescent microscope (Fig. 3A), we quantified the ratio of red/green fluorescent intensity using a microplate reader. Our data revealed that 2 h treatment of TNFα or H_2_O_2_ damaged *∆ψ*_***M***_ in AC16 cells, denoted by the decreased ratio of red/green fluorescent intensity in these cells compared to their vehicle control counterpart (Fig. 3B). Intriguingly, knockdown of Per2 further reduced TNFα- or H_2_O_2_-impaired *∆ψ*_***M***_ in AC16 cells compared to the control siRNA group (Fig. 3C), displaying a likely protective role of Per2 in maintaining mitochondrial function during stress. However, Per2 siRNA alone did not affect *∆ψ*_***M***_ (Fig. S3).

### Per2 knockdown increases cell death in AC16 human cardiomyocytes exposed to TNFα

**Figure 4:**
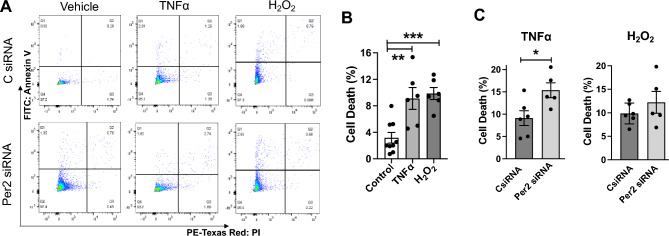
Per2 knockdown increased cell death in AC16 human cardiomyocytes exposed to TNFα or H_2_O_2_. (**A**) Representative flow cytometry images in AC16 cells transfected with control (C) or Per2 siRNA following 4 h treatment of TNFα or H_2_O_2_. (**B**) Either TNFα or H_2_O_2_ increased cell death in AC16 cells. (**C**) Per2 Knockdown potentiated cell death following TNFα. Mean ± SEM, *p < 0.05, **p < 0.01, ***p < 0.001.

TNFα or H_2_O_2_ can induce cell death in a variety of cells and mitochondrial membrane potential is also involved in apoptosis induction. Therefore, we evaluated the role of Per2 in regulating AC16 cell death following TNFα or H_2_O_2_ stimulation. After Annexin V and PI staining and analysis of Flow cytometry (Fig. 4A), our data indicated that 4 h treatment of TNFα or H_2_O_2_ significantly increased cell death in AC16 cells (Fig. 4B). Furthermore, Per2 knockdown resulted in greater cell death in TNFα-stressed AC16 cells compared to control.

siRNA transfected cells (Fig. 4C), suggesting that Per2 likely plays a role in TNFα-induced cell death signaling in cardiomyocytes. However, Per2 siRNA alone did not impact cell viability (Fig. S4).

### The implication of Per2 in mitochondrial respiration function following TNFα or H_2_O_2_ stress

**Figure 5:**
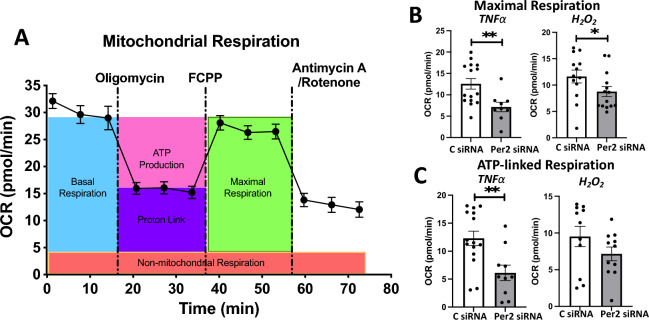
Role of Per2 in mitochondrial respiration function following TNFα or H_2_O_2_ stress. (**A**) Seahorse Cell Mito Stress Test assay in AC16 human cardiomyocytes shown as the OCR trace. (**B**) Calculated maximal respiration and (**C**) ATP-linked respiration in control or Per2 siRNA-transfected AC16 cells following 2 h treatment with TNFα and H_2_O_2_. Mean ± SEM, *p < 0.05, **p < 0.01. Dot represents individual wells.

**Figure 6:**
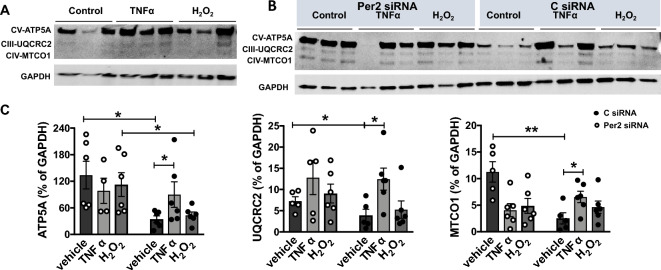
Role of Per2 in Oxphos levels in AC16 human cardiomyocytes following TNFα or H_2_O_2_ stress. (**A**) Oxphos expression [ATP5A-complex V (CV), UQCRC2-complex III (CIII), and MTCO1-complex IV (CIV)] in AC16 cells subjected to 4 h-TNFα or -H_2_O_2_. (**B**) Immunoblots of ATP5A, UQCRC2, and MTCO1 in AC16 cells transfected with Per2 or control siRNA following 4 h treatment with TNFα and H_2_O_2_. (**C**) Quantification of immunoblotting band intensity of ATP5A, UQCRC2, and MTCO1, represented as % of GAPDH. Mean ± SEM, *p < 0.05, **p < 0.01.

Our previous studies have demonstrated mitochondrial functional impairment in cardiomyocytes subjected to TNFα or H_2_O_2_^20,22^. In this study, we determined the role of Per2 in mitochondrial metabolic function in AC16 cells upon exposure of TNFα or H_2_O_2_ using Seahorse Cell Mito Stress test (Fig. 5A). While decreased Per2 level did not affect mitochondrial respiration function in AC16 cells without stress of TNFα or H_2_O_2_ (Fig. S5), Per2 knockdown did significantly decrease mitochondrial maximal respiration (Fig. 5B) and ATP-linked respiration (Fig. 5C) in AC16 cells subjected to 2 h treatment of TNFα or H_2_O_2_. We further studied effects of stress on mitochondrial oxidative phosphorylation (OXPHOS) complex proteins. Western blot data demonstrated that TNFα significantly increased complex V-ATP5A, complex III-UQCRC2 and complex IV-MTCO1 proteins in AC16 cells (Fig. 6A,C), while a trend of augmented levels of ATP5A and MTCO1 was noticed in AC16 cells exposed to H_2_O_2_. Per2 knockdown markedly upregulated protein levels of ATP5A and UQCRC2 and abolished TNFα-induced increase in these complex molecules (Fig. 6B,C).

## Discussion

Mitochondria are central to heart bioenergetics, as mitochondrial dysregulation is an underlying mechanism of cardiac dysfunction. In this present study, our data clearly demonstrated that: (1) acute stress reduced Per2 levels and concurrently impaired mitochondrial function in the heart mitochondria and adult cardiomyocytes; (2) Increased cell death and decreased $\Delta \psi$_M_ were observed in human cardiomyocytes treated with TNFα or H_2_O_2_; (3) Knockdown of Per2 potentiated TNFα-induced cell death and TNFα- or H_2_O_2_- disrupted $\Delta \psi$_M_; and (4) Per2 knockdown worsened TNFα- or H_2_O_2_-induced impairment of mitochondrial respiration function. These findings supported a strong implication of Per2 in mitochondrial regulation to protect cardiomyocytes during stress.

Evidence supports the local inflammation and redox imbalance as important primary factors in cardiomyocyte injury and cardiac functional damage^23–25^. Acute injury including ischemia induces excessive production of TNFα or H_2_O_2_ that leads to cell death (apoptosis and necrosis) in many types of cells. Our current findings confirmed that TNFα and H_2_O_2_ significantly increased cell death in human cardiomyocytes. However, it is unknown whether Per2 plays a role in TNFα- or H_2_O_2_-induced cell death in human cardiomyocytes.

In addition to being a circadian regulator protein, emerging research has suggested the involvement of Per2 in the regulation of cell death through several pathways. Per2 has been found to inhibit activation of PI3K/Akt signaling, thus reducing proliferation while promoting apoptosis in human adenocarcinoma cell lines^26^. In the cytosol, Per2 can function as a scaffolding protein to block mTORC1-induced cell proliferation and increase autophagy in the liver during fasting^27^. Our previous work has also shown that reduction of Per2 in H9c2 cells increased apoptosis following cold storage^10^. In this study, Per2 knockdown enhanced TNFα-induced cell death in human cardiomyocytes, suggesting that Per2 plays a role in the regulation of cell death in cardiomyocytes in response to local inflammatory mediators. TNFα binding to its receptors leads to cell death through multiple regulatory pathways including the extrinsic pathway of apoptosis involving mitochondria^19^. However, the interaction of Per2 and TNFα-initiated cell death pathways are not well elucidated and necessitates further study.

Of note, we did not observe the impact of Per2 in preventing cell death of human cardiomyocytes exposed to H_2_O_2_. Since only one time point (4 h treatment of H_2_O_2_) was studied, we cannot confirm whether the H_2_O_2_ treatment period is suitable to detect cell death here. It is evident that Per2 deficiency reduces the tolerance of H9c2 cells to H_2_O_2_ following serum shock, with increased cell death in Per2 knockdown H9c2 cells^28^. However, reduction of Per2 did not significantly affect cell death in the H9c2 cells only exposed to serum shock^28^. This suggests that the relationship between Per2 and cell death may vary depending on the different stressed conditions and the balance of various signaling pathways involved. The exact mechanisms through which Per2 impacts cardiomyocyte cell death are a subject of ongoing research. Future investigations are needed to completely understand the underlying mechanistic details of Per2’s role in modulating cell viability.

Recent studies have revealed that the clock regulator Per2 influences mitochondrial metabolic modulation^4–6^. Per2 regulates mitochondrial oxidative metabolism in mouse C2C12 myoblasts exposed to fatty acid oxidation^3^. During ischemia, Per2 is activated by the hypoxia-inducible factor1a (HIF-1a) pathway, which facilitates induction of glycolytic enzymes to maximize energy production through glycolysis in low O_2_ conditions^5^. Increased oxygen consumption and impaired glycolysis are observed in Per2KO mice subjected to myocardial ischemia. Disruption of Per2 affects mitochondrial morphology and glycogen accumulation following myocardial ischemia^5,8^. Our present findings indicate that Per2 knockdown significantly decreased mitochondrial maximal respiration and ATP-linked respiration in human cardiomyocytes exposed to TNFα or H_2_O_2_. However, the precise mechanisms through which Per2 influences mitochondrial metabolic regulation remains unknown. Emerging evidence has indicated that mutation of Per2 is reported to cause a decrease of complex I activity and a higher NADH/NAD + ratio, thus impacting mitochondrial activity^29^. In addition, Per2 binds to complex IV to mediate mitochondrial respiratory function in endothelial cells following hypoxia^11^. Indeed, the Western Blot data establishes dysregulation of complex III–V protein levels in the AC16 cells with Per2 knockdown, suggesting a potential disruption of the mitochondrial electron transport chain. This is a rational explanation of Per2 knockdown impairing mitochondrial respiratory capacity in human cardiomyocytes.

Notably, mitochondrial membrane potential is essential for preserving normal proton gradient, thus maintaining mitochondrial respiratory function. In this study, TNFα or H_2_O_2_ significantly reduced *∆ψ*_***M***_ in human cardiomyocytes. Knockdown of Per2 further worsened TNFα- or H_2_O_2_-impaired *∆ψ*_***M***_ compared to control counterparts. This disrupted *∆ψ*_***M***_ might be another reason to explain why Per2 is important in regulating mitochondrial metabolic function. In addition, severe decrease in mitochondrial membrane potential results in changes of mitochondrial structure to release cytochrome C, triggering intrinsic apoptosis through the activation of caspases^30^. This may also explain why reduction of Per2 enhances TNFα-induced cell death in cardiomyocytes.

Overexpression of Per2 via adenovirus provides heart protection from ischemia^31^. Daylight or intense light exposure increases cardiac Per2, leading to reduced myocardial damage following ischemia^5,32^. Intense light-upregulated Per2 overexpression also improves maintenance of endothelial barrier function via promotion of metabolic reprogramming of cellular adaptation to myocardial ischemia^11^. Considering the role of Per2 in modulating cell survival and mitochondrial function during stress from our current study and previous publications as discussed earlier, it is therefore logical that overexpression or delivery of Per2 is protective to prevent cell death in cardiomyocytes and ameliorate mitochondrial dysregulation following myocardia injury^5,33^, thus benefiting mitochondrial bioenergetics and cardiac function. We will explore the therapeutic potential of increasing cardiac Per2 to improve mitochondrial function in our future investigations.

**Figure 7:**
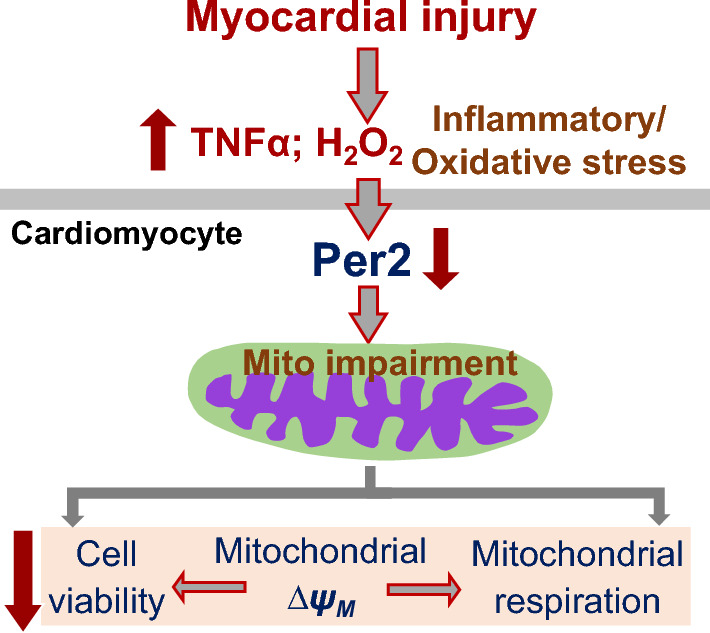
Per2 prevents mitochondrial damage in cardiomyocytes exposed to inflammatory and/or oxidative stress during myocardial injury.

In summary, our findings provide the direct evidence to support that Per2 is important to protect mitochondrial function and cell viability in human cardiomyocytes during inflammatory and oxidative stress (Fig. 7). We speculate that delivery of Per2 protein to cardiomyocytes during myocardial injury could serve as a cardioprotective factor, possibly by supporting mitochondrial function and improving cell survival observed in this study.

## Materials and methods

### Animals

All animal studies were conducted in compliance with the *Guide for the Care and Use of Laboratory Animals (*NIH Pub. No. 85-23, revised 1996). The animal protocol was reviewed and approved by the Institutional Animal Care and Use Committee of Indiana University. Male C57BL/6J mice (9–12 weeks) were purchased from the Jackson Laboratories (Bar Harbor, ME). The animals were acclimated for > 5 days with a standard diet feeding prior to the experiments and maintained on the same light–dark cycle. All animal experiments were performed between 10:00 am and 2:00 pm.

The study is reported in accordance with ARRIVE guidelines.

### Global myocardial ischemia in vivo

After deep anesthesia by isoflurane, the mice were placed supine on a heated pad (37 °C) and injected peritoneally with 0.15 ml of heparin (100 U/ml). The chest was then opened, and the diaphragm was dissected to introduce respiratory arrest. The heart was collected 30 min after the heart stopped beating (30 min-warm ischemia) and snap-frozen in liquid nitrogen. Freshly isolated mouse hearts without global myocardial warm ischemia served as control. Mitochondria were obtained by differential centrifugation using mitochondria isolation kit for tissue (ThermoFisher Scientific) according to manufacturer's protocols. The purity of mitochondrial preparations using this method was confirmed in our previous study^20^.

### Measurement of mitochondrial complex I activity

Mitochondrial complex I activity was detected using the enzyme activity microplate assay kit (Abcam, Cambridge, MA, ab109721) according to manufacturer's protocols. Isolated heart mitochondrial pellets (10 μg of mitochondrial protein) were resuspended in PBS with 10% detergent provided in the kit. Enzyme activities were analyzed by increased absorbance at OD = 450 nm due to the oxidation of NADH to NAD^+^ using a microplate reader (represented as absorbance changes per minute per milligram protein). Each sample was conducted in duplicate.

### Adult mouse cardiomyocyte isolation

By using a Langendorff perfusion system, cardiomyocytes were isolated from adult male mouse hearts based on our previous studies^20,22,34^. Briefly, after mice were heparinized and euthanized with isoflurane overdose, the hearts were excised and rapidly placed into ice-cold calcium-free perfusion buffer. Hearts were perfused and digested with collagenase II (1.5 mg/ml) in perfusion buffer containing 50 mM calcium. Isolated cardiomyocytes were then restored sequentially in perfusion buffer with calcium (100, 250, 500, or 1000 mmol/L CaCl_2_) and seeded into laminin (20 mg/ml)-precoated 6-well plate in cardiomyocyte plating medium. After 2-h cultivation for adherence, cardiomyocytes were treated with vehicle, 10 ng/ml TNF or 50 μM of H_2_O_2_ for 2 h and then collected for protein isolation. The doses of TNFα- and H_2_O_2_ were selected based on our previous studies^20,22^. The cardiomyocytes were also plated into laminin (20 mg/ml)-precoated XF96 cell culture plates with 1500 cells/well^22^. After the cultivation for adherence, the cells were used for the Seahorse XF Cell Mito Stress Assay.

### Knocking down of Per2 in AC16 human cardiomyocyte cell line

The AC16 human cardiomyocyte cell line was purchased from the Millipore Sigma and cultured in 100 mm cell culture dishes with DMEM/F12 (Sigma, D6434) containing 12.5% FBS (EMD Millipore, ES-009-B), 2 mM l-Glutamine (EMD Millipore, TMS-002-C), and 1X Penicillin–Streptomycin Solution (EMD Millipore, TMS-AB2-C) at 37 °C, 5% CO_2_ and 90% humidity, based on the manufacturer's protocols. AC16 cells were plated in 12-well plate at 1.0X10^5^ cells/well or in 96-well plate at 1.0X10^4^ cells/well. Twenty-four hour later, cells were transfected with human Per2 or control siRNAs (Life Technologies) using Lipofectamine 2000 (Life Technologies). After one day of transfection, normal AC16 growth medium was added. The cells were allowed to incubate for an additional day and then used for experiments. Different doses of Per2 siRNA (10, 20, and 30 pmol) were used to determine their effects on transcript levels of Per2 and the dose of 20 pmol was selected for subsequent trials in this study.

All cell culture studies were performed in accordance with our approved protocol by the Institutional Biosafety Committee of Indiana University.

### Real-time quantitative PCR

Total RNA was extracted from AC16 cells using Trizol (Life Technology) and used for the first-strand cDNA reverse transcription using Quantum (ThermoFisher Scientific). TaqMan assays were used to determine transcript levels of human Per2 (Fam dye-labeled) and GAPDH (VIC dye-labeled) (ThermoFisher Scientific) by Real-time PCR (7500 Real-Time PCR System, Applied Biosystems). The probes of both Per2 and GAPDH were added into the same reaction and Per2 expression was normalized to the GAPDH level.

### Western Blotting

The heart tissues and the cells were lysed in cold RIPA buffer containing Halt protease and phosphatase inhibitor cocktail (ThermoFisher Scientific). The protein and mitochondrial extracts were loaded into a 4–15% Criterion TGX Precast midi protein gel (Bio-Rad, Hercules, CA, USA) for electrophoresis and transferred to a nitrocellulose membrane. The membranes were incubated with the following primary antibodies respectively: Per2 antibody (ThermoFisher Scientific, 100107), OXPHOS antibody cocktail (complex I-NDUFB8, complex II-SDHB, complex IV-MTCO1, complex III-UQCRC2, and complex V-ATP5A) (ThermoFisher Scientific), and GAPDH (#2118) (Cell Signaling Technology, Beverly, MA, USA) and then incubated with the fluorescence-conjugated secondary antibody. A ChemiDoc system (BioRad) was used to detect immunoblotting bands, which were quantified using the Image J software (NIH).

### Cell death detection by flow cytometry

Control siRNA- and Per2 siRNA-transfected AC16 cells were collected from 12-well plate after treatment with vehicle, 100 ng/ml TNFα^35^ or 100 μM of H_2_O_2_^36^ for 4 h and stained with Annexin-V FITC and propidium iodide (PI) using a Dead Cell Apoptosis kit (BD Sciences). Dead cells were analyzed with a LSR4 flow cytometer (BD Biosciences) and determined by Flowjo software (Annexin-V + /PI− [apoptotic cells], Annexin V-/PI + [late apoptotic and necrotic cells] and Annexin V + /PI + [end stage apoptosis and death]). The experiments were repeated three times.

### Seahorse cell mito stress test

We detected mitochondrial bioenergetic response shown by oxygen consumption rate (OCR) in adult mouse cardiomyocytes and AC16 human cardiomyocytes using a Seahorse XF-96 instrument (Seahorse Biosciences, USA) as we recently reported^22,34^. Adult mouse cardiomyocytes (1500 cells/well) were treated with TNFα (10 ng/ml) or H_2_O_2_ (50 μM) in supplemented XF medium (5 mM Glucose, 1 mM pyruvate, and 2 mM Glutamine) for 1 h according to our previous studies^20,22^. AC16 cells (6000 cells/well) transfected with control or Per2 siRNAs were exposed to TNFα (100 ng/ml) or H_2_O_2_ (100 μM) in supplemented XF medium (25 mM Glucose, 1 mM pyruvate, and 2 mM Glutamine) for 2 h. The metabolic profile of these cells was detected sequentially as baseline OCR, ATP-linked respiration by adding oligomycin (Oligo, 1 μM), maximal uncoupled respiration after injecting FCCP (1 μM), and non‐mitochondrial respiration by addition of rotenone (R) and antimycin A (A) (1 μM). The basal OCR was calculated by subtracting non-mitochondrial OCR from the last value before addition of oligomycin. Maximal OCR was obtained by subtracting non-mitochondrial OCR from the FCCP-stimulated rate.

### Mitochondrial membrane potential

Two days after transfection with Per2 or control siRNA, AC16 human cardiomyocytes in the 96-well plate were treated with vehicle, 100 ng/ml TNFα or 100 μM of H_2_O_2_. Two-hour later, AC16 growth medium containing a fluorescent probe JC-1 (5 μM, G-Biosciences, St. Louis, MO, USA) was added. After 30-min incubation at 37C, live-cell imaging was taken using a Nikon Eclipse Ts2R microscope with a 20X objective. In addition, fluorescence intensity in each well was recorded using a microplate reader (BioTek) with total red (excitation: 535 nm; emission: 585 nm) and green (excitation: 485 nm; emission: 535 nm) fluorescence. Mitochondrial membrane potential was indicated by the red to green fluorescence intensity ratio.

### Statistical analysis

The results were means ± SEM and each dot represents individual measurement for each sample. Data was analyzed using either student *t*-test or one-way ANOVA. p < 0.05 indicates statistically significant difference. The GraphPad Prism software (GraphPad, La Jolla, CA, USA) was used for all statistical analyses.

### Supplementary Information

Supplementary Figures.

## Data Availability

The data presented in this paper are available by contacting Dr. Meijing Wang via meiwang@iupui.edu.
