# Supplementary material for: Importance of Per2 in cardiac mitochondrial protection during stress
Source: Sci Rep. 2024 Jan 14;14:1290. doi: 10.1038/s41598-024-51799-w (PMC10788343; doi:10.1038/s41598-024-51799-w)
Supplement: Supplementary file 1 — Supplementary Figures. [file 41598_2024_51799_MOESM1_ESM.pdf]

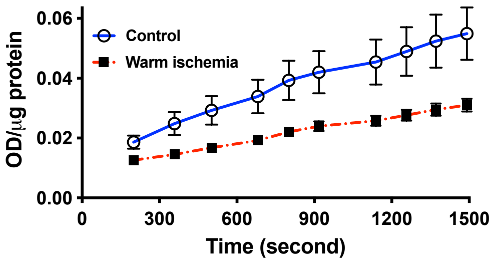

Figure S1. Changes of complex I activity along with time in male mouse hearts +/- 30-min warm ischemia. Mean +/- SEM, n = 4 mouse hearts per group.

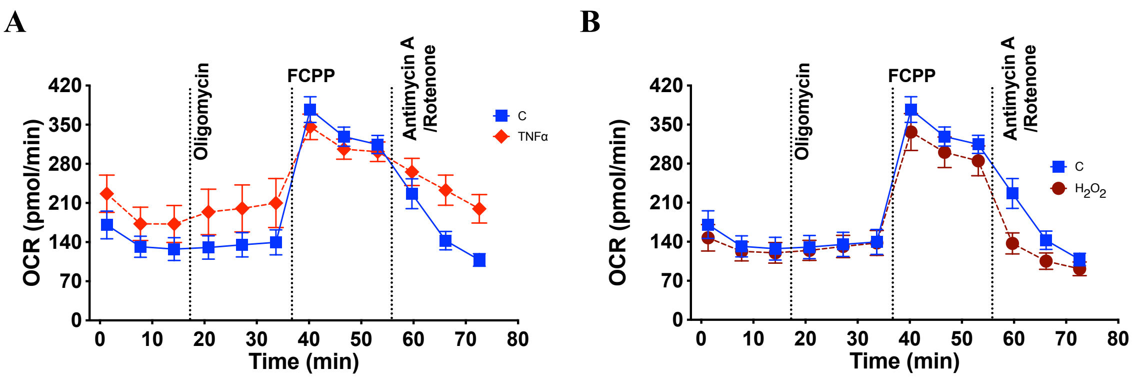

Figure S2. The OCR trace in adult mouse cardiomyocytes following vehicle, TNFα treatment (10 ng/ml) (A), or H_2O_2 (50 μM) (B).

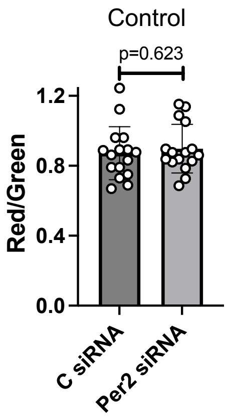

Figure S3. Mitochondrial membrane potential in AC16 cells transfected with control or Per2 siRNA without treatment of TNFα or H_2O_2.

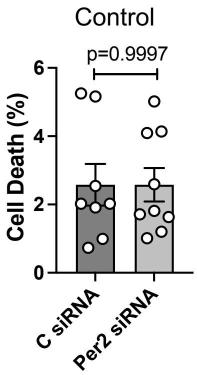

Figure S4. Cell death in AC16 cells transfected with control or Per2 siRNA without treatment of TNFα or H_2O_2.

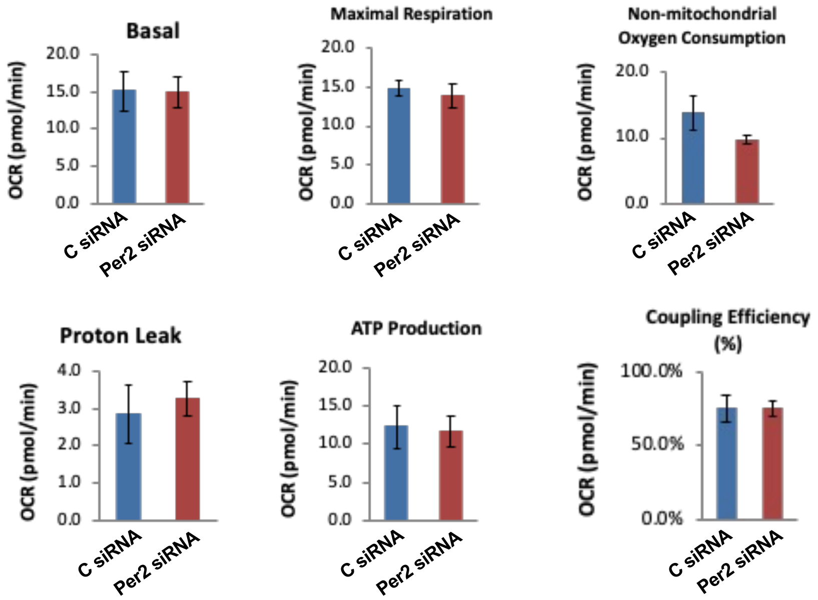

Figure S5. Metabolic profiling - calculated values for mitochondrial basal, maximal, non-mitochondrial, proton leak, ATP production and coupling efficiency parameters between control siRNA and Per2 siRNA transfected AC16 human cardiomyocytes without treatment of TNFα or H_2O_2.

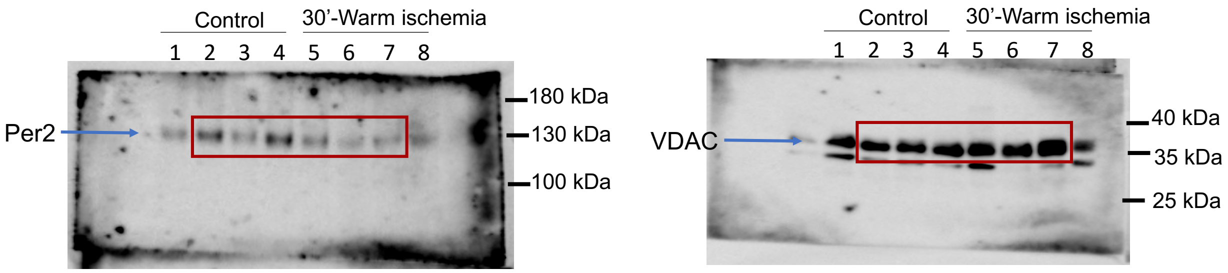

Figure S6. The original Western blots for figure 1B.

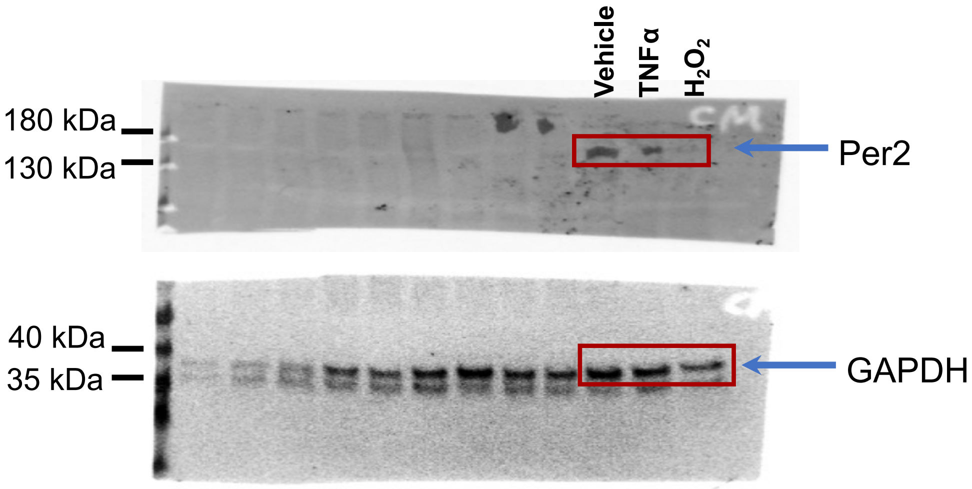

Figure S7. The original Western blots for figure 1D.

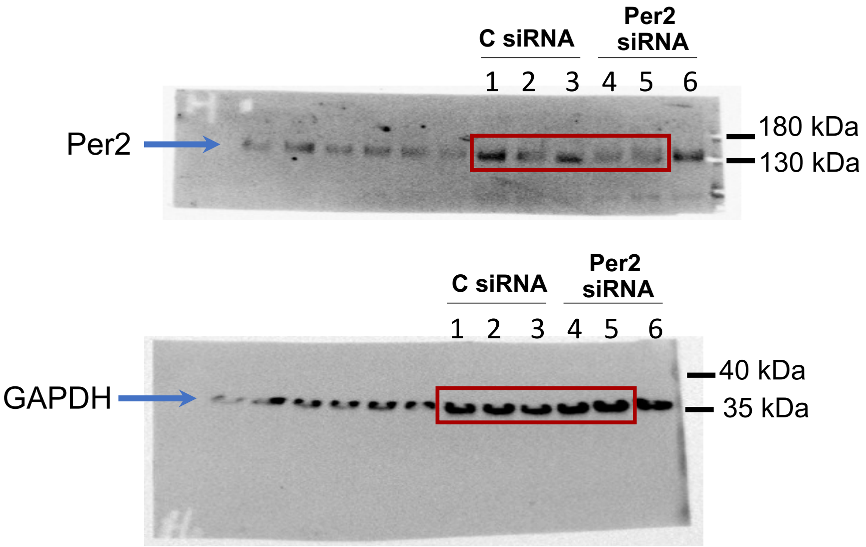

Figure S8. The original Western blots for figure 2C.

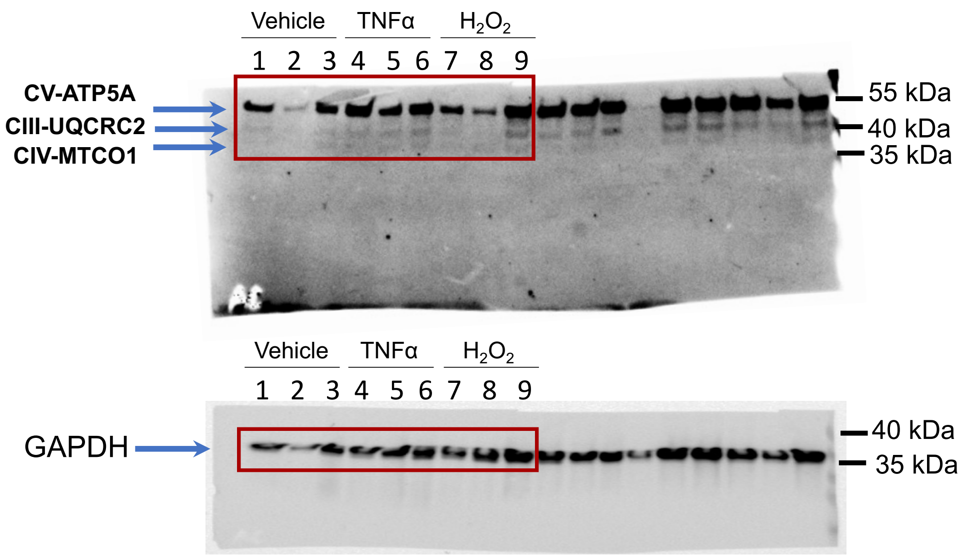

Figure S9. The original Western blots for figure 6A.

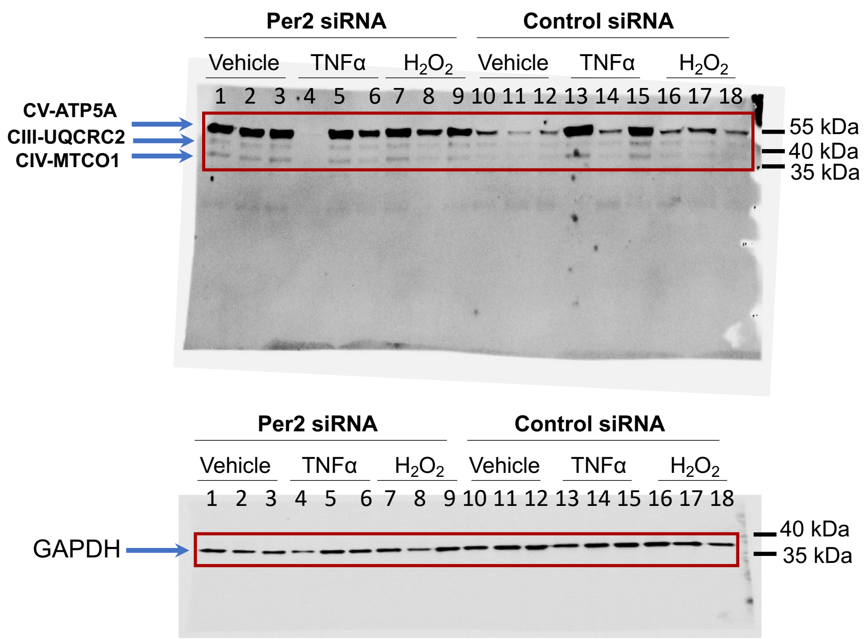

Figure S10. The original Western blots for figure 6C.
